# Supplementary material for: Causal Relationship between Aging and Anorexia Nervosa: A White-Matter-Microstructure-Mediated Mendelian Randomization Analysis
Source: Biomedicines. 2024 Aug 16;12(8):1874. doi: 10.3390/biomedicines12081874 (PMC11351342; doi:10.3390/biomedicines12081874)
Supplement: Supplementary file 1 [file biomedicines-12-01874-s001.zip › Supplementary Figure/Figure S4/Singe SNP plot of MR analysis for TL to ubm-b-1597.pdf]

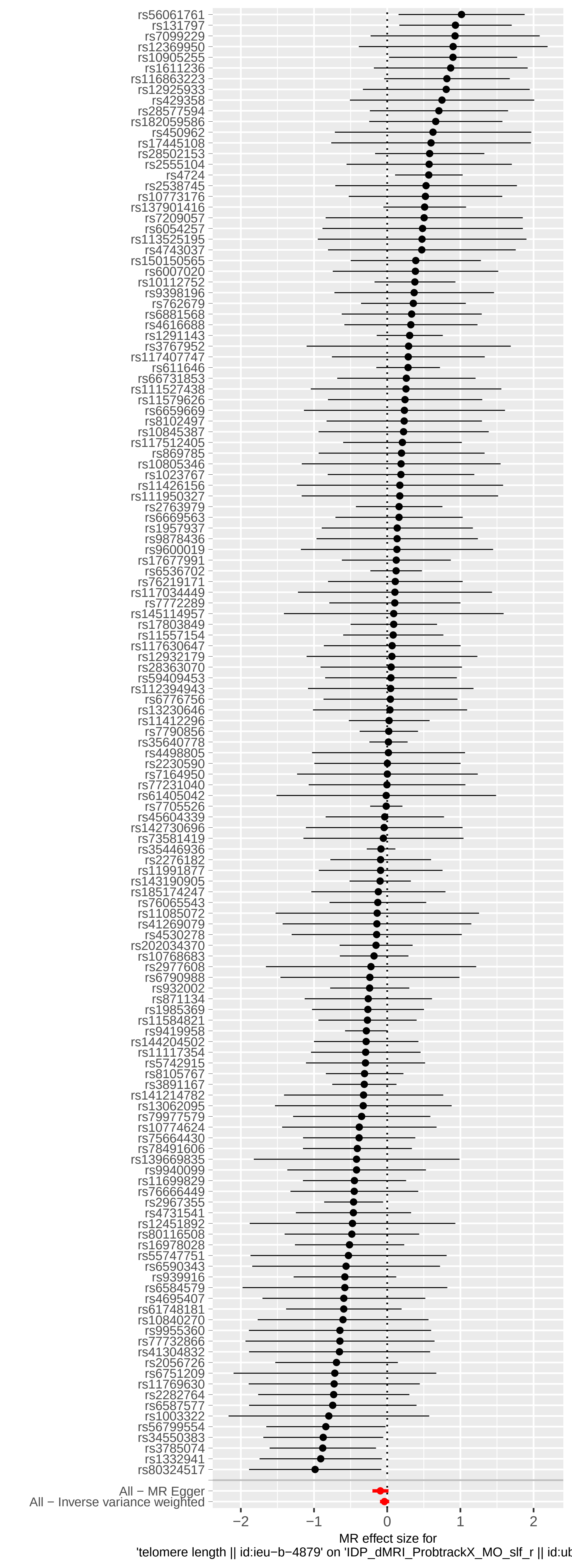

rs56061761
rs131797
rs7099229
rs12369950
rs10905255
rs1611236
rs116863223
rs12925933
rs429358
rs28577594
rs182059586
rs450962
rs17445108
rs28502153
rs2555104
rs4724
rs2538745
rs10773176
rs137901416
rs7209057
rs6054257
rs113525195
rs4743037
rs150150565
rs6007020
rs10112752
rs9398196
rs762679
rs6881568
rs4616688
rs1291143
rs3767952
rs117407747
rs611646
rs66731853
rs111527438
rs11579626
rs6659669
rs8102497
rs10845387
rs117512405
rs869785
rs10805346
rs1023767
rs11426156
rs111950327
rs2763979
rs6669563
rs1957937
rs9878436
rs9600019
rs17677991
rs6536702
rs76219171
rs117034449
rs7772289
rs145114957
rs17803849
rs11557154
rs117630647
rs12932179
rs28363070
rs59409453
rs112394943
rs6776756
rs13230646
rs11412296
rs7790856
rs35640778
rs4498805
rs2230590
rs7164950
rs77231040
rs61405042
rs7705526
rs45604339
rs142730696
rs73581419
rs35446936
rs2276182
rs11991877
rs143190905
rs185174247
rs76065543
rs11085072
rs41269079
rs4530278
rs202034370
rs10768683
rs2977608
rs6790988
rs932002
rs871134
rs1985369
rs11584821
rs9419958
rs144204502
rs11117354
rs5742915
rs8105767
rs3891167
rs141214782
rs13062095
rs79977579
rs10774624
rs75664430
rs78491606
rs139669835
rs9940099
rs11699829
rs76666449
rs2967355
rs4731541
rs12451892
rs80116508
rs16978028
rs55747751
rs6590343
rs939916
rs6584579
rs4695407
rs61748181
rs10840270
rs9955360
rs77732866
rs41304832
rs2056726
rs6751209
rs11769630
rs2282764
rs6587577
rs1003322
rs56799554
rs34550383
rs3785074
rs1332941
rs80324517
All − MR Egger
All − Inverse variance weighted
−2
−1
0
1
2
MR effect size for
'telomere length || id:ieu−b−4879' on 'IDP_dMRI_ProbtrackX_MO_slf_r || id:ub
